# Design of a low-delay 4-bit parallel prefix adder using QCA technology

**DOI:** 10.1038/s41598-025-04742-6

**Published:** 2025-07-01

**Authors:** Tushar Niranjan, Anirban Nayak, Sreehari Veeramachaneni, Syed Ershad Ahmed

**Affiliations:** 1 Department of Electrical and Electronics Engineering, Birla Institute of Technology and Science, Pilani, Hyderabad Campus, Jawahar Nagar, Kapra Mandal, Medchal District, Hyderabad, Telangana 500078 India; 2 Department of Information Technology, Sri Sivasubramaniya Nadar College of Engineering, Chennai, 603110 India

**Keywords:** Parallel prefix adder, QCA, VLSI, Electrical and electronic engineering, Computational nanotechnology

## Abstract

This paper presents a novel low-delay 4-bit Parallel Prefix Adder (PPA) implemented as a multilayer circuit using Quantum Dot Cellular Automata (QCA) technology. PPAs are among the most suitable architectures for high-speed digital design, offering significant advantages in scalability and performance over traditional Ripple Carry Adders (RCAs) and Carry Flow Adders (CFAs). The proposed design provides a fast, compact, ergonomic, and energy-efficient alternative to QCA adders adopting these architectures. This work enhances existing PPA modules, including XOR gates, Half Adders, Black Modules, and Gray Modules, by tailoring them to optimally fit the core PPA structure. The proposed PPA achieves a 26% reduction in cell count, a 31% reduction in area and a 57% reduction in delay compared to existing PPA designs. Utilizing a hybrid crossover methodology, the design reduces delay by 25% relative to the fastest 4-bit QCA adder reported in the literature and lowers the area-delay cost by 11% compared to the most economical design. Simulated using the QCADesigner-E Version 2.2 software, the proposed adder demonstrates energy dissipation comparable to existing designs, solidifying its practicality and efficiency for high-speed QCA-based applications.

## Introduction

Binary adders are fundamental components in modern digital systems, performing essential arithmetic operations that power everything from basic processors to advanced computing architectures. As our world becomes increasingly reliant on high-speed, low-power computation, the efficiency of these adders directly impacts the performance and energy consumption of devices. In traditional CMOS technology, scaling limitations are becoming more pronounced, pushing the need for alternative design approaches. Quantum-dot Cellular Automata (QCA)^1^ presents a promising solution, offering the potential for faster, smaller, and more power-efficient circuits. Designing binary adders within the QCA framework is therefore critical to meet the demands of next-generation computing technologies.

This article focuses on integrating the high-performance capability of Parallel Prefix Adders (PPAs) with the design flexibility of QCA to address common PPA challenges, such as dense and complex interconnections, that hinder existing designs. By harnessing the strengths of both the architecture and the technology, this study aims to transform the approach to designing PPA circuits using QCA. The specific contributions of this work are highlighted below:

- Development of compact arithmetic modules in QCA, specifically tailored for the PPA architecture, with optimizations focused on minimizing overall cell count and maximizing area efficiency.
- Implementation of a hybrid crossover methodology, leveraging selective use of crossovers to minimize delay and achieve the overarching goal of superior speed.
- Optimization of the floorplan through targeted refinements in module design and clocking configuration, with strategic component alignment enhancing design ergonomics, all while maintaining signal integrity, resulting in enhanced overall circuit coherence.

The preliminaries of QCA technology are outlined in “Preliminaries of QCA technology” section, followed by a thorough review of literature on QCA adders in “Related works” section. “The parallel prefix adder” section presents an overview of the arithmetic foundation of Parallel Prefix Adders. “Proposed work and contributions” section presents an in-depth examination of the proposed design and thoroughly details the contributions of this work. A detailed discussion of the key results and observed trends is provided in “Results and discussions” section, and “Conclusion” section concludes with insights into potential directions for future research in this domain.

## Preliminaries of QCA technology

This section provides foundational insights by introducing four C’s in QCA-Cells, Clocking, Crossovers, and Cost- that underpin the proposed design and methodology in subsequent sections.

### Cells in QCA

**Fig. 1:**
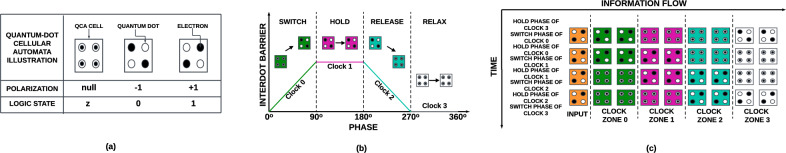
(**a**) Standard QCA cell configurations (**b**) clock phases in QCA (**c**) clock zones in QCA.

Quantum-Dot Cellular Automata (QCA) cells are the fundamental building blocks of QCA-based circuits, consisting of four quantum dots arranged in a square configuration. Each cell confines two electrons that can tunnel between adjacent dots while remaining within cell boundaries. Due to Coulombic repulsion, these electrons occupy diagonally opposite dots, minimizing electrostatic interaction energy. This spatial arrangement establishes the cell’s polarization, representing binary states ‘0’ and ‘1’. Neighboring cell interactions enable one cell’s polarization to influence adjacent cells, facilitating binary information transmission without conventional current flow and offering a novel approach to nanoscale digital circuit design^2^. Figure 1a shows standard QCA cell configurations: the unpolarized state and the two possible polarization states, − 1 and +1 respectively.

### Clocking in QCA

In QCA circuits, the clocking mechanism is essential for controlling information flow through sequential clock phases and zones. Figure 1b illustrates the four phases each QCA cell undergoes-switch, hold, release, and relax-to manage signal timing and integrity. In the switch phase, the cell’s potential barrier lowers, allowing polarization adjustment based on neighboring interactions. The hold phase then raises this barrier, locking in the cell’s polarization to preserve the signal, while the release phase lowers it again, enabling depolarization. Finally, the relax phase resets the cell to its initial state for the next cycle^3^. Each cell starts at a specific clock phase, moving through these steps to ensure synchronized progression across cycles. As shown in Fig. 1c, these phases are organized across clock zones-spatial regions within the circuit where cells operate in the same phase simultaneously.

Practical implementation of QCA circuits in the real world demands the use of dedicated clocking circuitry to facilitate state transitions and charge manipulation throughout the design. The architecture of clocking circuits is continually evolving, with methods like the Efficient, Scalable, Regular (ESR) clocking scheme^4^, emerging as a leading approach due to its advantages in design flexibility, energy efficiency, cost-effectiveness, and manufacturability. Accordingly, designing circuits that are compatible with contemporary clocking schemes is crucial to maintain viability for fabrication.

### Crossovers in QCA

**Fig. 2:**
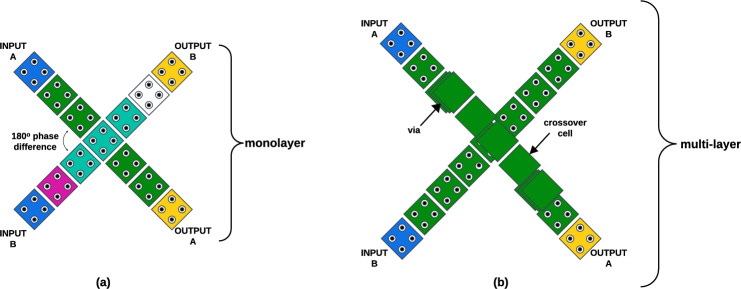
Crossovers in QCA (**a**) coplanar (**b**) multilayer.

Crossovers, or wire-crossings, in QCA are achieved through two main approaches. In coplanar crossovers, signals intersect within the same layer; to prevent signal corruption, the crossing cells must maintain a 180-degree phase difference, as shown in Fig. 2a. This approach can reduce cell count and area; however, it increases delay due to the need for additional clock phases. Alternatively, multilayer crossovers, illustrated in Fig. 2b, route signals over or under each other using additional layers, which avoids adding delay but requires extra cells and area. These crossover strategies offer design flexibility, allowing QCA circuits to balance performance, area, and fabrication complexity.

### Cost in QCA

A variety of cost functions have been proposed in the literature to evaluate the optimality of QCA circuits, from general models such as the Area-Delay Cost Function, shown in Eq. (1), to more specialized formulations such as the Majority-Based QCA-Specific Cost Function^5^ in Eq. (2).

1 $$\begin{aligned} & {\text {Cost}_{\text {Area-Delay}} = A \times T^2} \end{aligned}$$

2 $$\begin{aligned} & \quad {\text {Cost}_{\text {QCA}} = \left[ (M_3 + F \times M_5)^K + I + C^L\right] \times T^P} \end{aligned}$$

(where A - Area, T - Delay, M3 - Number of three-input majority gates, M5 - Number of five-input majority gates, F - Ratio of number of cells of the five-input majority under consideration to the number of cells of the three-input majority gate, I - Number of inverters, C - Number of crossovers, K,L,P - Exponential Weightings)

This study adopts the Area-Delay Cost Function from Eq. (1) as it concisely captures both resource usage and latency, with particular emphasis on the latter, given the focus on minimizing delay. It also reflects the relationship between the two parameters, where signal transmission relies on cell-to-cell interactions, making larger circuits with longer pathways naturally slower in propagation. However, this function does not consider factors influencing manufacturability. For instance, multilayer crossovers significantly increase fabrication complexity^6^. Additionally, an important aspect regarding the fabrication cost is the number of fixed-polarity inputs used, which require supplementary QCA circuitry^7^. To present a comprehensive view of overall cost, a qualitative discussion of these factors is included in the results and discussion section.

## Related works

Ripple Carry Adders (RCAs) remain the most extensively researched architecture in QCA literature, with numerous designs proposed. Among coplanar designs, the RCA developed by Chugh and Singh^8^ achieves the lowest delay but it is also the only coplanar RCA to use fixed inputs which add manufacturing overhead. The design by Sasamal et al.^9^ has the best area-delay cost in the category. Coplanar RCAs by Ramesh and Rani^10^ and by Abedi et al.^11^ are well balanced and very similar in terms of QCA metrics. Ramesh et al.^12^ propose a coplanar RCA for BCD additions which achieves the best area efficiency in the category. Other coplanar RCA implementations include those by Balali et al.^13^, Hönninen and Takala^14^, Kassa et al.^15^ and Qureshi et al.^16^. Within the existing literature on QCA adders, regardless of architecture, the multilayer RCA by Roshany and Rezai^17^ is considered the most optimized in terms of cell count and cost, though its multilayer structure complicates fabrication. De and Das^18^ propose a well balanced RCA which is also the only multilayer RCA to use a fixed polarity input, taking on manufacturing overhead. The design proposed by Mohammadi et al.^19^ rivals it in terms of delay whereas the one proposed by Sridharan and Pudi^20^ rivals it in terms of cell count and cost. Other multilayer RCA implementations of interest can be found in the works of Hashemi et al.^21^ and Cho and Swartzlander^22^. However, RCAs generally struggle with signal congestion and scaling in larger circuits rendering them less suitable for high-speed applications.

Carry Save Adders (CSAs) have also seen a few QCA implementations, though they face significant optimization challenges. The design by Erniyazov and Jeon^23^ suffers from increased delay due to coplanar limitations. Cho and Swartzlander^22^ have proposed a multilayer pipelined design. A recent design by Seyedi et al.^24^ proposes a fault tolerant multilayer CSA implementation for IOT Devices.

Carry Look-Ahead Adders (CLAs) offer unique advantages but encounter trade-offs similar to CSAs in QCA. The coplanar CLA design by Cho and Swartzlander^25^ simplifies the floorplan but limits delay reduction and wiring congestion, reducing its applicability in high-speed contexts. A modern design has been put forth by Qureshi et al.^16^ which improves the cell count and area, but trades-off further on latency for practicality. Among multilayer designs, the CLA design by Erniyazov and Jeon^23^ presents a cost-friendly alternative, but it still struggles to efficiently scale delay reduction compared to other architectures. The design proposed by Cho and Swartzlander^22^ and the one proposed by Ahmadpour^26^ measure similar in performance but the latter avoids fabrication overheads due to fixed inputs.

Carry Flow Adders (CFAs) are a niche option in QCA, providing a balance between speed and design complexity. The designs proposed by Roohi et al.^27^ and Cho and Swartzlander^28^ demonstrate competitive performance; however, CFAs generally require more area and complex clocking schemes, which impact scalability, particularly for high-speed applications.

Sridharan and Pudi^29^ propose efficient coplanar and multilayer implementations of a Hybrid Adder that integrates the Ladner-Fischer and RCA architectures. Despite these advantages, hybrid designs suffer from interconnection complexity, resulting in larger area usage and a more intricate clocking structure, which increases cost.

Leveraging parallelism to significantly reduce computational delay, PPAs are a compelling choice for high-speed applications. In existing QCA implementations, Sridharan and Pudi^20^ propose several designs of PPA such as Brent-Kung, Kogge-Stone and Ladner-Fischer which are well optimized in terms of area and cost, although their complex structures introduce potential fabrication and signal integrity challenges. The work by Touil et al.^30^, notable for its superior delay performance, requires trade-offs in area and cell count, affecting cost-effectiveness and scalability. Thanos and Vergos^31^ propose a scalable alternative. This study proposes a novel PPA that builds on these foundations, aiming to overcome the latency limitations of previous designs through a hybrid crossover methodology and to serve as a suitable building block for high-speed applications.

## The parallel prefix adder

An evolution of the CLA, the PPA architecture is the focus of this study. It is favored for its ability to deliver rapid and efficient computations, particularly in large-scale circuits where minimizing computational delay is essential. By utilizing a tree-like structure to compute carry bits in parallel, the PPA architecture outperforms adders that rely on serial carry propagation, such as the RCA, making it highly effective for time-sensitive applications. This scalable architecture is also an ideal fit for adder design in QCA, as the technology can help alleviate the dense interconnection challenges typically burdening traditional PPAs.

**Fig. 3:**
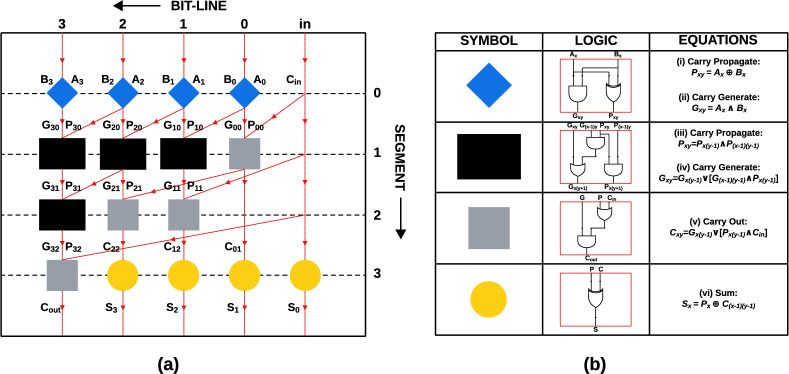
(**a**) Simplified tree graph of 4-bit PPA architecture (**b**) the PPA modules.

Figure 3a provides a simple tree graph of the 4-bit PPA architecture^32^ clearly highlighting the various modules and signals in play. To adopt a standard nomenclature for intermediary signals, the circuit is placed on a grid system with bit-lines running vertically and segment lines running horizontally. Bit-lines correspond to specific bit positions and propagate signals vertically, while segments represent computation stages, organizing modules horizontally. All the adder modules are positioned at nodes within this grid. In Fig. 3b, the subscript x denotes the bit-line associated with the module from which the signal originates, while the subscript y refers to the segment of the circuit where the module resides. For example, $G_{21}$ refers to a carry-generate signal from a module located on bit-line 2, segment 1. Peripheral signals are only associated with a bit-line, without reference to a segment. In addition, the grid includes an input-line that runs parallel to the least significant bit-line.

Segment 0 in Fig. 3a consists of four blue diamond-shaped modules, each representing a half-adder (HA). The architecture processes two 4-bit binary inputs, $A_3A_2A_1A_0$ (generalized as $A_x$) and $B_3B_2B_1B_0$ (generalized as $B_x$), along with a single-bit carry-in ($C_{in}$). Each corresponding bit of these input vectors is directed to a half-adder module, where the logical operations (i) and (ii) specified in Fig. 3b are executed. The sum is then routed to the carry propagate wire ($P_{xy}$), while the carry is directed to the carry generate wire ($G_{xy}$).

In Segment 1 of Fig. 3a, three black rectangular modules and one gray square module are present. The black module produces carry-generate and carry-propagate signals whereas the gray module complements this function by generating intermediary carry signals that eventually lead to the carry-out signal. Each black module located on the x-th bit-line of Segment 1 receives the carry-generate and carry-propagate signals from both the *x*-th and *(x-1)*-th bit-lines of Segment 0. These modules generate the corresponding carry-generate and carry-propagate signals for the subsequent segment. Meanwhile, the gray module positioned on the *0*-th bit-line utilizes the carry-generate and carry-propagate signals from the same bit-line of Segment 0, in addition to the carry-in ($C_{in}$) from the input line, to produce the $C_{01}$ carry signal. The logic structures of the black and gray modules are depicted in Fig. 3b and are defined by equations (iii), (iv), and (v) shown therein.

In Segment 2 of Fig. 3a, one black module and two gray modules are present. The black module receives the current carry-generate and carry-propagate signals from Segment 1 and propagates the next generation of these signals. Concurrently, the gray modules use the current carry-in signal, along with the other carry signals, to produce the next generation of the carry-out signal, which will be used in Segment 3.

In Segment 3 of Fig. 3a, in addition to a gray module, there are four yellow circular modules, each representing an exclusive-OR (XOR) gate. The gray module is tasked with generating the final carry-out signal ($C_{out}$) by processing the incoming carry-generate, carry-propagate, and carry-in signals. Each XOR module computes a single bit of the final sum by utilizing the relevant carry-propagate and carry-in signals. The standard XOR logic for each yellow module is illustrated in Fig. 3b, with the corresponding equation provided as equation (vi) in the same figure. It should be noted that the color scheme used for the modules in Fig. 3 is consistently applied in Fig. 5 to clearly demarcate module boundaries within the 4-bit QCA PPA circuit.

In existing literature, the few successful implementations of PPAs in QCA fail to fully exploit the performance potential of its architecture. Due to suboptimal routing and inefficient module designs, previous circuits have a high cell count and mediocre latency. This study aims to address these shortcomings by capitalizing on the merits of QCA technology, as detailed in the following section.

## Proposed work and contributions

This study aims to achieve high-speed circuit performance within the innate constraints of QCA technology through the proposed multilayer 4-bit PPA design, illustrated in Fig. 5. The proposed work introduces the following circuit design contributions.

### Compact module designs tailored for PPA architecture

The proposed QCA architecture employs functional units meticulously designed and optimized from the outset to minimize cell count, reduce area footprint, and achieve minimal delay.

#### The modified half-adder module

**Fig. 4:**
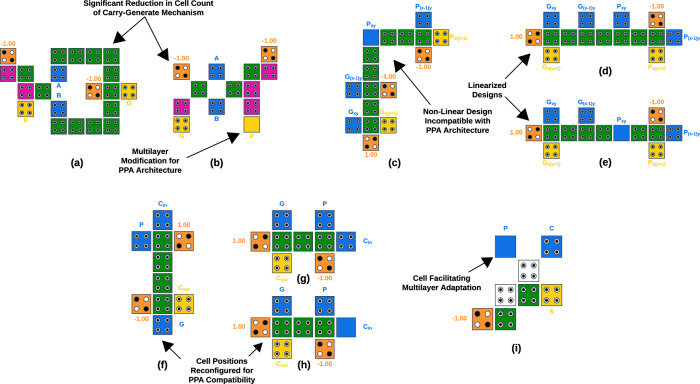
QCA modules (**a**) half adder^34^ (**b**) proposed modified half adder (**c**) black module^30^ (**d**) proposed coplanar linearized black module (**e**) proposed multilayer linearized black module (**f**) gray module^30^ (**g**) proposed coplanar reconfigured gray module (**h**) proposed multilayer reconfigured gray module (**i**) optimal XOR module adapted for PPA.

The modified half-adder module, depicted in Fig. 4b, consists of 13 cells and occupies an area of 0.019 $\upmu {{m}}^{2}$ as measured using QCADesigner-E Version 2.2^33^. It operates across two phases of the QCA clock to generate the required intermediate output signals. Since one of these phases serves as the inherent rest phase, the circuit contributes a net delay of 0.25 units. In comparison, the original half-adder design shown in Fig. 4a, as proposed by Safaiezadeh et al.^34^, operates within a strictly coplanar paradigm. This earlier design employs 21 cells, resulting in a larger area of 0.024 $\upmu {{m}}^{2}$. The proposed half-adder achieves a significant reduction in cell count and area-38% fewer cells and 21% less area-due to its compact carry-generation mechanism. Additionally, the proposed design introduces a multilayer adaptation that enhances its compatibility with the PPA architecture. The inclusion of a vertical interconnect cell, represented as a solid cell in Fig. 4b, facilitates connections between different layers and enables crossover functionality. By combining a compact carry-generation mechanism with a multilayer architecture, this implementation achieves a refined balance of design simplicity, area efficiency, and overall performance. It stands out as one of the most optimized QCA half-adder designs to date.

#### The linearized black module

**Fig. 5:**
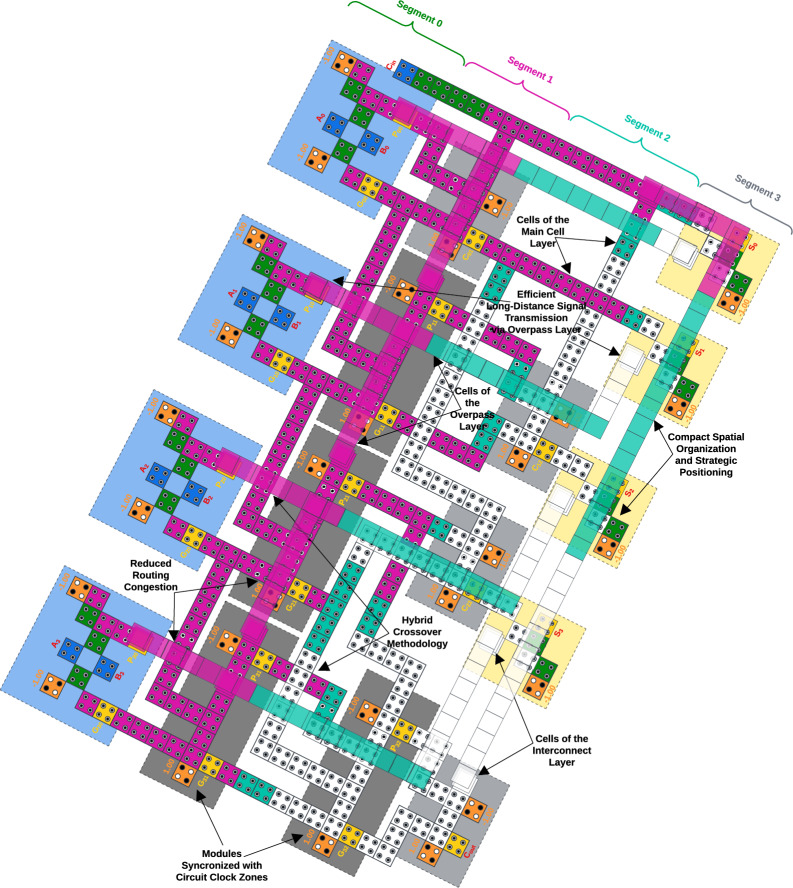
A detailed illustration of the proposed 4-bit QCA parallel prefix adder circuit.

The black module proposed by Touil et al.^30^ features a non-linear design as shown in Fig. 4c. However, the parallelized nature of components in the PPA architecture-such as input streams and computation stages, as depicted in Fig. 4c-creates alignment challenges when integrating this non-linear design into the QCA PPA circuit. These misalignments significantly increase the cell count due to the additional cells required for connecting wires and crossovers. To address these challenges, the proposed black module linearizes the design presented in paper^30^, eliminating alignment issues while preserving its full functionality. As illustrated in Fig. 4d & e, both coplanar and multilayer versions of the linearized black module are utilized in the same multilayer circuit, depending on the orientation of the input signals in each specific instance. The module consists of 15 cells, occupies an area of 0.024 $\upmu {{m}}^{2}$, and operates within a single phase of the QCA clock. Operating in the same phase as its input signals, this module introduces no additional delay to the circuit. The linear arrangement ensures direct alignment between the module’s input and output cells with adjacent components, as well as proper integration within the overall architecture which is evident in Fig. 5. This reduces the reliance on connecting cells and coplanar crossovers, thereby minimizing cell count and improving overall performance.

#### The reconfigured gray module

The gray module is another non-standard logic unit utilized in PPA architectures, complementing the black module. While the design proposed by Touil et al.^30^, shown in Fig. 4f, is optimized for cell count and performance, its integration into the PPA architecture is hindered by the configuration of individual cells within the compact module. The proposed design addresses this limitation by exchanging the positions of the carry-generate input cell and the fixed logic 1 input cell. This minor reconfiguration enables the module to be placed horizontally instead of the vertical orientation presented by Touil et al.^30^. This alignment adheres to the architectural principle of parallel signal paths in PPA, significantly reducing the additional cells required for wiring from inconvenient directions. Moreover, a redundant cell located at the center of the module is eliminated, further optimizing the design. The reconfigured gray module consists of 9 cells, occupying an area of 0.017 $\upmu {{m}}^{2}$. Similar to the black module, both coplanar and multilayer configurations of this module, as shown in Figure 4g & h, are used in the same multilayer circuit. The module operates within a single phase of the QCA clock, aligning with the phase of its input signal, thereby avoiding any contribution to circuit delay. This reconfigured gray module is among the most compact components in the proposed design, achieving exceptional area efficiency while maintaining full functionality.

#### The adapted exclusive-OR module

The exclusive-OR (XOR) module consists of 8 cells and covers an area of 0.016 $\upmu {{m}}^{2}$. The design shown in Fig. 4i is a multilayer adaptation of the XOR gate architecture proposed by Safaiezadeh et al.^34^. Similar to the black and gray modules, this unit operates within a single phase of the QCA clock, synchronized with its input phase. The block is strategically oriented within the circuit, as shown in Fig. 5, to minimize redundant cell usage and reduce unnecessary crossovers, enhancing overall design efficiency.

### Hybrid crossover methodology for superior performance

The traditional PPA architecture^32^ is characterized by intricate interconnections necessary for achieving computational parallelism-a feature that serves as both a boon and a bane. While this parallelism facilitates high-speed computation, in QCA design, the resulting routing complexity introduces numerous coplanar crossovers, significantly increasing delay. Minimizing delay is crucial for achieving high-speed performance in QCA circuits. Fortunately, QCA technology itself offers a pathway to resolve this inherent challenge.

In PPAs, as depicted in Fig. 3b, signals like carry-in ($C_{in}$) and carry propagate ($P_{xy}$) are essential inputs at multiple stages of the circuit. Rather than routing these signals through the main cell layer, shown in Fig. 5, an alternative approach is adopted and multilayer crossovers are utilized. Specific connections are accommodated in an auxiliary layer, depicted in Fig. 5 using translucent cells, which runs above and parallel to the main cell layer. This auxiliary layer is termed the ‘Overpass Layer’ due to its role in reducing traffic congestion. A third layer of cells, termed the ‘Interconnect Layer’ and shown in Fig. 5, connects the main cell layer and the overpass layer at specific junctions where signals are transferred between them. The mindful usage of these multilayer crossovers not only helps save on delay but also contributes to making the circuit more compact through better spatial organization. It should be noted that the coplanar crossovers in Segment 2 are retained. Relocating these crossovers to an auxiliary layer is constrained by two primary factors:

- Implementing these crossovers in the overpass layer, would necessitate coplanar crossovers with propagation wires that already traverse this layer, thus negating the intended benefits.
- Utilizing an underpass layer would result in an extended clock zone for Segment 1, risking excessive cell density. Within long wires, it is important to keep a check on the number of cells placed in the same clock zone. This is necessary to prevent signal back propagation and reduce noise^22^.

Parallel Prefix Adders implementing this hybrid-crossover methodology will have an overall delay given by Eq. (3), where $n$ is the bit-width. The term $\log _2(n)-1$ denotes the number of stages in the parallel prefix tree needed to compute all carry signals. The constant term $0.25$ corresponds to the reduced delay introduced by Segment 0, or the modified half-adder stage, while $0.5$ represents the constant delay due to coplanar crossovers—a reduced number compared to traditional PPA architectures—due to multilayer crossovers of the hybrid crossover methodology. The variable $s$ is a scaling factor that accounts for additional clock zones required to prevent signal corruption^22^.

3 $$\begin{aligned} \text {Delay(n)} = 0.5s \cdot \left( \log _2(n) - 1 \right) + 0.25 \end{aligned}$$

The scaling factor is 1 for a 4-bit PPA, resulting in an overall delay of 0.75, and it increases as $n$ increases. The complete circuit with all the layers stacked together is provided in Fig. 5. The proposed design therefore adopts a hybrid approach to implementing crossovers, leveraging the flexibility of QCA, by selectively employing coplanar crossovers and multilayer crossovers to attain superior performance.

### Optimized floorplan for enhanced circuit coherence

In circuit design, achieving optimal performance often hinges on subtle, yet impactful refinements. After thorough consideration, a series of strategies have been implemented to optimize the circuit floorplan, as outlined below:

- Different segments of the circuit, including the various modules, have been configured to run as per their designated clock zones to establish a pipelined flow of information. This is observed through the uniform transition in the colours of the cells as one moves from the inputs to the outputs of the circuit. Careful accommodation of the modules so as to run on the minimum number of clock phases possible helps achieve maximum timing efficiency.
- In QCA circuits, signal transmission relies on direct cell-to-cell interactions, with longer pathways naturally leading to slower signal propagation. To address this and to reduce risks of signal back-propagation and noise, each clock zone is limited to a maximum of 15 cells^22^.
- All elements are positioned as closely as possible, maintaining a minimum gap of one cell between components to ensure structural integrity and signal isolation. This strategy reduces voids and achieves a compact design optimized for area, while also reducing energy dissipation and easing fabrication.
- The carry-in ($C_{in}$) signal is routed above the XOR units to enable parallel feeding, demonstrating the strategic positioning of functional units to enhance circuit coherence and achieve compactness.

These targeted design refinements collectively improve the QCA-based PPA, achieving an optimal balance of performance, compactness, and reliability within this specialized architecture.

## Results and discussions

The proposed circuit was designed and simulated using QCADesigner-E Version 2.2^33^. An extended version of QCADesigner 2.0.3.^35^, QCADesigner-E remains the leading tool for both academic and industrial research in QCA circuit design. All the circuits from the references cited use QCADesigner for design and simulation.

**Fig. 6:**
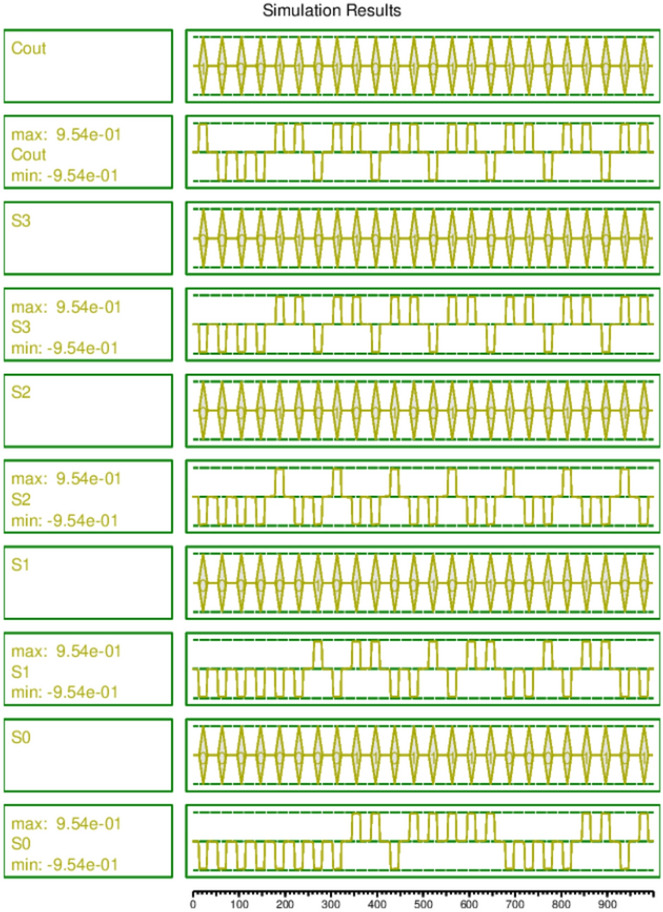
Simulation results of the proposed 4-bit QCA PPA circuit.

Figure 6 shows the simulation waveforms of the output stream of the circuit consisting of $S_3S_2S_1S_0$ and $C_{out}$. The circuit generated stable and accurate output against both directed as well as random testing. Standard QCA cell dimensions were maintained across all three layers, with each cell having a width and height of 18 nm and a dot diameter of 5 nm. The Coherence Vector Simulation Engine was utilized for simulating the circuit, with energy dissipation tracking enabled.

This study uses the Area-Delay Cost Function instead of a QCA Specific Cost Function, as the proposed design doesn’t employ conventional majority gates or inverters as building blocks. The shortcomings of the former with respect to multilayer crossovers and other fabrication parameters like fixed inputs are addressed qualitatively.

**Fig. 7:**
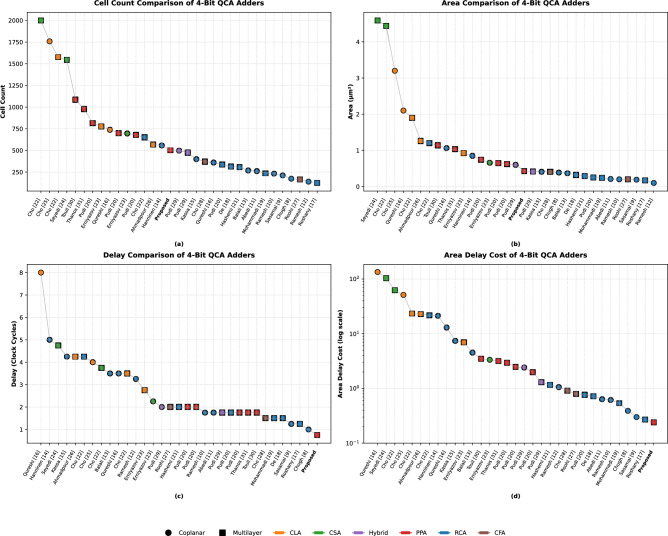
Comparison of 4-bit QCA adders (**a**) cell count (**b**) area (**c**) delay (**d**) cost.

The proposed 4-bit QCA PPA achieves a total cell count of 503, representing a 26% reduction compared to the next-best PPA design presented by Sridharan and Pudi^20^, which adopts the Brent-Kung architecture. This notable improvement stems primarily from the meticulous selection of modules and their targeted modifications to align with the PPA architecture. The use of these customized modules eliminates redundant routing and significantly reduces the number of cells employed. Furthermore, a reduction in cell count at the module level-enabled by the enhanced carry-generation mechanism in the half-adder module-also contributes to this optimization. With a circuit area of 0.43 $\upmu {{m}}$^2^, the proposed design achieves a 31% reduction compared to the Ladner-Fischer PPA reported by Sridharan and Pudi^20^. This reduction in circuit area is largely attributed to the decreased cell count. Additionally, strategic design optimizations significantly enhance spatial and area efficiency. Key improvements include routing the carry-in signal over XOR gates, arranging modules to minimize voids within the circuit layout, and maintaining a single-cell spacing between circuit elements wherever feasible. These refinements collectively contribute to a highly compact and efficient design.

The proposed PPA outperforms all existing 4-bit QCA Adders in terms of circuit delay, as shown in Figure 7c. With a delay of 0.75 units, it is the fastest 4-bit QCA adder reported in the literature, surpassing the RCA presented by Chugh et al.^8^ by 25%. With a margin of 57 %, it surpasses the delay achieved by existing PPA implementations such as the Brent-Kung PPA proposed by Sridharan and Pudi^20^, the fast Ling-Carry PPA proposed by Thanos and Vergos^31^, and the Kogge-Stone PPA proposed by Touil et al.^30^. This remarkable performance is primarily attributed to the hybrid crossover methodology, which strategically combines coplanar and multilayer crossovers to minimize redundant coplanar connections, significantly reducing delay. Additionally, the use of optimally designed modules with minimal delay further contributes to this achievement. Aligning the modules with their respective clock zones, as illustrated in Fig. 5, helps pipeline this effort. Lastly, the design achieves an 11% reduction in area-delay cost compared to the most economical RCA design reported by Roshany et al.^17^ and an 88% reduction in area-delay cost compared to the most economical PPA in existing literature. Figure 7d helps visualize this evaluation metric on a logarithmic scale. These savings are of great significance especially when compared with other multilayer designs, as they have comparable fabrication costs. This reduction is directly attributable to the minimization of delay, which has an exponential impact on area-delay cost.

However, the proposed PPA has the following shortcomings. Although it surpasses all CSAs, CLAs, and other PPAs in terms of cell count and area, it trails behind RCAs, CFAs, and hybrid designs, as shown in Fig. 7a and b respectively. This is justified, as minimizing cell count and area was not the primary objective of this design. Furthermore, the PPA architecture is inherently not optimized for size and area, unlike architectures such as RCA. Instead, it is designed for speed, and the proposed design demonstrates this objective effectively. A major shortcoming of the proposed design arises from a fabrication perspective. Multilayer crossovers are difficult to fabricate, but they are an integral part of this design methodology and therefore contribute to increased manufacturing costs. Furthermore, the use of fixed inputs in the custom modules requires additional QCA wiring to be laid out beneath the main cell layer, adding fabrication overhead. Table 1 compares the proposed design with a comprehensive set of coplanar and multilayer 4-bit QCA adders from the literature. The total energy dissipation of the proposed PPA is only 0.171 eV, making it a fast, compact, ergonomic, and energy-efficient 4-bit QCA adder design with considerable scope for improvement in fabrication aspects.

**Table 1:** A comprehensive comparison of existing 4-bit adders in QCA.

Layers	Type	Reference	Fixed polarities	Cell count	Area ($\upmu {{m}}^{2}$)	Delay (clk cycles)	AT^2^-Cost
Coplanar	CLA	Qureshi^16^	+1 and − 1	739	2.10	8.00	134.40
		Cho^25^	+1 and − 1	1758	3.20	4.00	51.20
	CSA	Erniyazov^23^	− 1	696	0.66	2.25	3.34
	Hybrid	Pudi^29^	+1 and − 1	498	0.60	2.00	2.41
	RCA	Hanninen^14^	None	558	0.85	5.00	21.25
		Kassa^15^	None	401	0.41	4.25	7.41
		Balali^13^	None	269	0.37	3.50	4.53
		Qureshi^16^	None	361	1.06	3.50	12.98
		Ramesh^12^	None	140	0.10	3.25	1.06
		Ramesh^10^	None	234	0.20	1.75	0.62
		Abedi^11^	None	262	0.21	1.75	0.64
		Sasamal^9^	None	212	0.19	1.25	0.30
		Chugh^8^	+1 and − 1	174	0.39	1.00	0.39
Multilayer	CFA	Roohi^27^	None	165	0.20	2.00	0.8
		Cho^28^	None	371	0.41	1.50	0.91
	CLA	Ahmadpour^26^	None	568	1.26	4.25	22.76
		Cho^22^	+1 and − 1	1575	1.90	3.5	23.23
		Erniyazov^23^	+1 and − 1	777	0.92	2.75	6.96
	CSA	Seyedi^24^	+1 and − 1	1542	4.59	4.75	103.56
		Cho^22^	+1 and − 1	1999	4.44	3.75	62.44
	Hybrid	Pudi^29^	+1 and − 1	475	0.42	1.75	1.30
	RCA	Cho^22^	None	651	1.20	4.25	21.68
		Hashemi^21^	None	308	0.29	2.00	1.16
		Pudi^20^	None	339	0.25	1.75	0.77
		Mohammadi^19^	None	237	0.24	1.50	0.54
		De^18^	− 1	314	0.32	1.50	0.72
		Roshany^17^	None	125	0.17	1.25	0.27
	PPA	Pudi^20^	+1 and − 1	698	0.62	2.00	2.47
		Pudi^20^	+1 and − 1	815	0.74	2.00	2.95
		Pudi^20^	+1 and − 1	680	0.65	1.75	1.98
		Thanos^31^	+1 and − 1	977	1.03	1.75	3.17
		Touil^30^	+1 and − 1	1085	1.14	1.75	3.49
		**Proposed**	**+1 and − 1**	**503**	**0.43**	**0.75**	**0.24**

## Conclusion

The era of post-CMOS alternatives is drawing closer. Ensuring state-of-the-art designs for fundamental arithmetic components like adders is essential to facilitate a smooth transition. Efficient and well-optimized designs will serve as the backbone for adapting more complex architectures, enabling the full potential of emerging technologies like QCA. The development of this 4-bit PPA design in QCA represents a foundational step toward building higher bit width components, such as 32-bit or 64-bit adders, which will be indispensable in realizing high-performance, next-generation computing systems.

## Data Availability

All data generated or analysed during this study are included in this paper.
